# Age-Related Changes in Pain Perception Are Associated With Altered Functional Connectivity During Resting State

**DOI:** 10.3389/fnagi.2020.00116

**Published:** 2020-05-07

**Authors:** Ana M. González-Roldán, Juan L. Terrasa, Carolina Sitges, Marian van der Meulen, Fernand Anton, Pedro Montoya

**Affiliations:** ^1^Cognitive and Affective Neuroscience and Clinical Psychology, Research Institute of Health Sciences (IUNICS) and Balearic Islands Health Research Institute (IdISBa), University of the Balearic Islands (UIB), Palma, Spain; ^2^Institute for Health and Behaviour, University of Luxembourg, Luxembourg, Luxembourg

**Keywords:** aging, resting-state, functional connectivity, pain perception, pain-related network

## Abstract

Aging affects pain experience and brain functioning. However, how aging leads to changes in pain perception and brain functional connectivity has not yet been completely understood. To investigate resting-state and pain perception changes in old and young participants, this study employed region of interest (ROI) to ROI resting-state functional connectivity (rsFC) analysis of imaging data by using regions implicated in sensory and affective dimensions of pain, descending pain modulation, and the default-mode networks (DMNs). Thirty-seven older (66.86 ± 4.04 years; 16 males) and 38 younger healthy participants (20.74 ± 4.15 years; 19 males) underwent 10 min’ eyes-closed resting-state scanning. We examined the relationship between rsFC parameters with pressure pain thresholds. Older participants showed higher pain thresholds than younger. Regarding rsFC, older adults displayed increased connectivity of pain-related sensory brain regions in comparison to younger participants: increased rsFC between bilateral primary somatosensory area (SI) and anterior cingulate cortex (ACC), and between SI(L) and secondary somatosensory area (SII)-(R) and dorsolateral prefrontal cortex (PFC). Moreover, decreased connectivity in the older compared to the younger group was found among descending pain modulatory regions: between the amygdala(R) and bilateral insula(R), thalamus(R), ACC, and amygdala(L); between the amygdala(L) and insula(R) and bilateral thalamus; between ACC and bilateral insula, and between periaqueductal gray (PAG) and bilateral thalamus. Regarding the DMN, the posterior parietal cortex and lateral parietal (LP; R) were more strongly connected in the older group than in the younger group. Correlational analyses also showed that SI(L)-SII(R) rsFC was positively associated with pressure pain thresholds in older participants. In conclusion, these findings suggest a compensatory mechanism for the sensory changes that typically accompanies aging. Furthermore, older participants showed reduced functional connectivity between key nodes of the descending pain inhibitory pathway.

## Introduction

Pain in older adults is poorly understood. Aging seems to be associated with increased pain thresholds and poor functioning of endogenous pain inhibition mechanisms (Lautenbacher, 2012; Lautenbacher et al., 2017). Altogether, this seems to indicate that older adults would activate the pain system later than younger ones, thus showing signs of pain insensitivity, but the relative lack of pain inhibition would lead to pain escalation over time, thus showing more prevalent pain symptoms (Lautenbacher, 2012). However, although published findings of age-related abnormalities at the psychophysical level seem robust, functional changes in the central nervous system that are possibly causing or maintaining these increased thresholds and the commented inhibitory deficits are still unknown (Farrell, 2012).

Age-related changes in the spontaneous organization of the brain have been linked to the cognitive, perceptual and motor alterations that frequently accompany aging (Ferreira and Busatto, 2013; Madden et al., 2017; King et al., 2018; Solesio-Jofre et al., 2018). In this sense, previous studies have found that older participants displayed enlarged cortical representations of the body and enhanced cortical excitability of primary somatosensory cortex (SI), leading to reduced tactile perception (Shaffer and Harrison, 2007; Kalisch et al., 2009; Lenz et al., 2012; Catley et al., 2014). Moreover, it has been shown that brain areas such as the prefrontal cortex (PFC), amygdala (AMY) and insula (INS) undergo a significant reorganization of resting-state functional connectivity (rsFC) with aging (He et al., 2017; Xiao et al., 2018). These brain regions play a key role in acute pain processing and several studies have shown a strong relationship between rsFC of INS, AMY and anterior cingulate cortex (ACC) with pain perception (Boly et al., 2007; Proverbio et al., 2009; Ploner et al., 2010), empathy for pain (Vaidya and Gordon, 2013) and psychologically induced stress (van Marle et al., 2010; Vaisvaser et al., 2013). Furthermore, a large number of studies have shown that chronic pain is also associated with changes in rsFC between brain regions involved in affective, sensory and descending modulatory pain processing [namely ACC, INS, AMY, thalamus (THA), PFC, SI and secondary somatosensory regions (SII), and the periaqueductal gray (PAG); Cifre et al., 2012; Kong et al., 2013; Kucyi and Davis, 2015; González-Roldán et al., 2016]. Hence, it is likely that cerebral changes described above may also be related to the alterations in pain perception in the older population, as well as to their greater vulnerability to suffering from chronic pain disorders (Farrell, 2012). However, most of the literature on rsFC and pain is from younger participants, and no studies have examined rsFC and pain in healthy older adults.

Therefore, the present study aimed to analyze the impact of aging on pain processing (pain pressure thresholds) and associated rsFC among sensory, affective and descending modulatory pain processing structures. Moreover, considering the last studies suggesting that the default mode network (DMN) is also active during experimental pain tasks in both young (Kong et al., 2010) and older adults (Monroe et al., 2015), the possible contribution of this network in age-related pain perception changes was also examined. We hypothesized that older, as compared to younger participants, would display higher pain thresholds, together with enhanced functional connectivity of somatosensory cortices and reduced connectivity between those regions involved in pain inhibition.

## Materials and Methods

### Participants

Participants were recruited from the University of the Balearic Islands (the older group was recruited from a senior program of the University or University employees). The sample was composed of 37 healthy older adults (16 men; 66.86 ± 4.04, the age range of 60–79 years) and 38 healthy young adults (19 men; 20.74 ± 4.15, the age range of 18–26 years; see Table 1 for sociodemographic characteristics).

**Table 1:** Sociodemographic and clinical data of younger and older groups.

		Younger (*n* = 38)	Older (*n* = 32)	Statistic	*p*
Age (years)		20.74 (2.34)	66.84 (4.15)	*t*_(68)_ = 58.35	**0.001**
Sex (males)		19	14	$\chi_{\left(1,70\right)}^{2}$ = 0.27	0.602
Educational level				$\chi_{\left(1,70\right)}^{2}$ = 6.66	**0.036**
	<8	0	3		
	8–12	1	4		
	>12	37	25		
Medication	Anxiolytic	0	1		
	Antidepressant	0	5		
	Anti-inflammatory	1	4		
	Cholesterol	0	22		
	Hypertension	0	15		
	Hypoglycemic	0	3		
	Others	7	27		
Finger pain threshold (N)		57.80 (24.67)	78.18 (23.55)	*t*_(68)_ = 3.51	**0.001**
Wrist pain threshold (N)		44.76 (21.23)	55.30 (18.88)	*t*_(68)_ = 2.18	**0.033**
Shoulder pain threshold (N)		42.81 (17.80)	56.15 (24.39)	*t*_(68)_ = 2.64	**0.010**
Finger (0–100 pain rating)		31.04 (19.86)	50.16 (24.11)	*t*_(68)_ = 3.64	**0.001**
Wrist (0–100 pain rating)		34.84 (18.63)	51.41 (23.03)	*t*_(68)_ = 3.33	**0.001**
Shoulder (0–100 pain rating)		30.76 (17.76)	48.75 (22.70)	*t*_(68)_ = 3.72	**0.001**
Blood pressure (mmHg)	Systolic	120.03 (15.25)	131.66 (15.07)	*t*_(68)_ = 3.19	**0.002**
	Diastolic	73.95 (9.43)	77.22 (17.90)	*t*_(68)_ = 0.98	0.332
PHQ-9		3.49 (2.80)	2.56 (2.71)	*t*_(65)_ = −1.39	0.176
GAD-7		3.45 (3.37)	3.28 (3.30)	*t*_(68)_ = −0.21	0.836
PANAS	Positive	32.50 (6.21)	37.09 (6.28)	*t*_(68)_ = 3.07	**0.003**
	Negative	12.42 (2.62)	13.34 (3.59)	*t*_(68)_ = 1.24	0.219

*Mean (Standard Deviation) and t-student comparisons are showed (significant differences in bold, *p* < 0.05). N, Newton; PHQ-9, Patient Health Questionnaire; GAD-7, Generalized Anxiety Disorder Assessment; PANAS, Positive and Negative Affect Schedule*.

All participants were interviewed in a previous screening session to exclude those who presented any of the following criteria: any current psychiatric or neurological condition, acute or chronic pain, uncontrolled hypertension, history of drug abuse, cognitive impairment (operationalized as a score below 27 in the Mini-Mental State Examination; Lobo et al., 1999), or left-handed (assessed by the Edinburgh Handedness Inventory; Oldfield, 1971). All individuals were naive to the experiment and gave informed consent after the experimental procedure was explained. The study was conducted following the Declaration of Helsinki (1991) and was approved by the Ethics Committee of the Balearic Islands (ref.: IB 3429/17 PI).

### Questionnaires

Before the day of the main experiment, all participants underwent an interview to assess clinical characteristics through a health interview and self-report questionnaires. They completed the Spanish versions of the Patient Health Questionnaire (PHQ-9; Kroenke et al., 2001) and the Generalized Anxiety Disorder Assessment (GAD-7) questionnaire (Garcia-Campayo et al., 2010). Finally, the Positive and Negative Affect Schedule (PANAS; Watson et al., 1988) was also filled out to assess participants’ moods during the experiment.

### Measurement of Pressure Pain Thresholds

First, to control confounding variables, blood pressure was measured in the right arm with a tensiometer (OMRON MX2, OMRON Healthcare, Hoofddorp, Netherlands) after the participant was seated and after they had rested for 5 min. Then, pressure pain thresholds were assessed always by the same experimenter and applying a previously used procedure (Martínez-Jauand et al., 2013a,b; Riquelme et al., 2016; Terrasa et al., 2018). Pressure pain thresholds were measured three times following the method of limits with a manual digital dynamometer using a flat rubber tip (1 cm^2^, Force One, Wagner Instruments, Greenwich, CT, USA) at three body locations of the non-dominant body site: index fingertip, medial area of the ventral surface of the wrist and dorsal area of the shoulder, measured in counterbalanced order. All subjects were specifically instructed to indicate when the pressure became painful. Stimulation stopped just when this point was reached. The subjects were informed that the investigation was aimed at determining the pain threshold, and not pain tolerance. Therefore, the pressure pain threshold was defined as the mean of the amount of pressure in Newtons (N) at which participants perceived the pressure stimulus as painful in the two last assessments for each location. Participants were asked to rate the subjective pain sensation of the stimulus on a 100-point numerical scale (0: no pain, 100: maximum pain).

### Brain Imaging Acquisition

After the measurement of pressure pain thresholds, all participants underwent an MRI and fMRI scanner on a GE 3T scanner (General Electric Signa HDx, GE Healthcare, Milwaukee, WI, USA) at the Son Espases University Hospital. For each participant, 240 whole-brain echo-planar images were acquired over 10 min with the eyes closed [36 transversal slices per volume; 3 mm slice thickness; 90° flip angle; repetition time (TR): 2,500 ms; echo time (TE): 35 ms; 64 × 64 matrix dimensions; 240 mm field of view; 3.75 × 3.75 × 3 mm voxel size]. The structural imaging data consisted of T1-weighted images. Twenty-five participants were acquired with the following parameters: 292 slices per volume; repetition time (TR): 7.84 s; echo time (TE): 2.976 ms; matrix dimensions, 256 × 256; 256 mm field of view; 1 mm slice thickness; 12 flip angle. Fifty participants were acquired with the following parameters: 220 slices per volume; TR: 7.9 s; TE: 3 ms; matrix dimensions, 256 × 256; 256 mm field of view; 1 mm slice thickness; 12 flip angle. T1 imaging data was only used to perform intraindividual coregistration and nuisance pre-processing. Scanner noise was passively reduced by using in-ear hearing protection. Also, foam cushions were placed over the ears to restrict head motion and further to reduce the impact of scanner noise.

### Brain Imaging Analyses

The connectivity analyses were performed with the CONN-fMRI Fc toolbox v18a (Whitfield-Gabrieli and Nieto-Castanon, 2012) and with SPM 12 (Wellcome Department of Imaging Neuroscience, London, UK^1^). All the structural and functional sequences were pre-processed using the CONN’s default pipeline for volume-based analysis: resampling to 2 × 2 × 2-mm voxels and unwarping, centering, slice time correction, normalization to the Montreal Neurological Institute (MNI) template, outlier detection (ART-based scrubbing) and smoothing to an 8-mm Gaussian kernel. Motion parameters (translations in the x, y and z directions) were used as multiple regressors and images with motion over 2.0 mm were regressed entirely out of the time course. To ensure data quality, any participant with more than 20% of the scans removed were excluded from the analyses (five of the older participants, two men). Furthermore, blood-oxygen-level-dependent (BOLD) data underwent a denoising process, including the regression of white matter and cerebrospinal fluid signals by using the CompCor method (Behzadi et al., 2007) in a single linear regression step. Finally, a band-pass filter (0.01–0.09 Hz) was applied to reduce noise effects and low-frequency drift.

To examine changes in functional connectivity within DMN structures and within pain-network areas due to aging, two separate region of interest (ROI) to ROI analyses were performed. DMN main regions were provided by the CONN toolbox and were originally derived from ICA analyses based on the human connectome project (HCP) dataset of 497 subjects. These regions included medial prefrontal cortex (mPFC), posterior cingulate cortex (PCC)/precuneus and bilateral lateral parietal (LP). Second, based on previous fMRI studies on experimental pain in patients with chronic pain and healthy controls (Gracely et al., 2002; Tracey and Mantyh, 2007; Zaki et al., 2007; Cifre et al., 2012), 14 ROIs from the pain-related network were selected. Concretely, bilateral INS, AMY and THA, as well as, ACC were defined with masks from the Harvard-Oxford Atlas. Bilateral dorsolateral prefrontal cortex (dlPFC), SI and SII were defined as described by Monroe et al. (2017) from the following Brodmann Areas (BA): dlPFC = BA 9 and 46, SI = BA 1, 2 and 3, and S2 = BA 40 and 44. Finally, as described by Coulombe et al. (2017), PAG was defined with a 6 mm sphere centered on MNI coordinates *x* = 0, *y* = −32, *z* = −12. Table 2 shows the MNI coordinates of each used ROI. Then, individual correlation maps were generated extracting the mean resting-state BOLD time course from all the ROIs and calculating the correlation coefficients between the BOLD time-course of each pair of ROIs. Correlations were obtained by applying the general lineal model (GLM) and bivariate correlation analysis weighted for haemodynamic response function (HRF) were obtained and used as rsFC measures. Finally, one-way Analysis of covariance (ANCOVAs) using sex as covariate were performed to examine group differences (*p* < 0.05 false discovery rate-corrected) in rsFC measures.

**Table 2:** Center of Montreal Neurological Institute coordinates for each region of interest (ROI) within the default-mode network and within the pain network, extracted from an anatomical atlas and previous studies.

ROI	*x*	*y*	*z*
mPFC	1	55	−3^a^
LP (R)	47	−67	29^a^
LP (L)	−39	−77	33^a^
PCC/precuneus	1	−61	38^a^
ACC	1	18	24^b^
INS (R)	37	3	0^b^
INS (L)	−36	1	0^b^
AMY (R)	23	−4	−18^b^
AMY (L)	−23	−5	−18^b^
THA (R)	11	−18	7^b^
THA (L)	−10	−19	7^b^
dlPFC (R)	38	34	30^c^
dlPFC (L)	−37	34	30^c^
SI (R)	43	−28	53^c^
SI (L)	−41	−29	53^c^
SII (R)	52	−37	37^c^
SII (L)	−51	−37	37^c^
PAG	0	−32	−12^d^

*mPFC, the medial prefrontal cortex; LP, lateral parietal; PCC, posterior cingulate cortex; SI, primary somatosensory cortex; SII, secondary somatosensory cortex; ACC, anterior cingulate cortex; dlPFC, dorsolateral prefrontal cortex; INS, insula; AMY, amygdala; PAG, periaqueductal gray matter; THA, thalamus; L, Left; R, Right. ^a^Human Connectome Project 497-subjects ICA analyses, ^b^Harvard-Oxford Atlas, ^c^Monroe et al. (2017), ^d^Coulombe et al. (2017)*.

Furthermore, we wanted to assess if changes in connectivity values were related to changes in pain perception. For this purpose, subjective pain ratings and pressure pain threshold indexes were computed by averaging the three-body locations (finger, wrist, and shoulder). Pearson’s correlations were computed between rsFC showing significant differences between groups and the pain threshold indexes by using SPSS (IBM Corp. Released 2015. IBM SPSS Statistics for Windows, Version 23.0. Armonk, NY, USA: IBM Corp). Finally, given that PANAS positive scores were higher in older in comparison to younger participants (see below), these scores were also correlated to rsFC scores. The new *p*-value considered as significant after Bonferroni Correction for multiple comparisons was <0.003 (resulting from *p* = 0.05/17 functional connectivity measures).

### General and Psychophysical Analyses

Group differences in sex and educational level were analyzed with Chi-Square Tests. Age, questionnaire scores, systolic and diastolic blood pressure, pressure pain thresholds and related subjective pain ratings were analyzed with Student *t*-tests. These analyses were conducted using SPSS (IBM Corp. Released in 2015. IBM SPSS Statistics for Windows, Version 23.0. Armonk, NY, USA: IBM Corp).

## Results

### Sociodemographic and Questionnaire Data

Statistical analyses of sociodemographic and questionnaires data (Table 1) showed that the older group had a lower educational *level* ($\chi_{\left(1,70\right)}^{2}$ = 6.66, *p* = 0.038), higher systolic blood pressure (*t*_(68)_ = 3.19, *p* = 0.002) and higher Positive dimension of PANAS (*t*_(68)_ = 3.07, *p* = 0.003) when compared to the younger group. No group differences were found regarding sex, diastolic blood pressure or other mood measures (PHQ-9, GAD-7, Negative dimension of PANAS).

### Pressure Pain Thresholds

**Figure 1:**
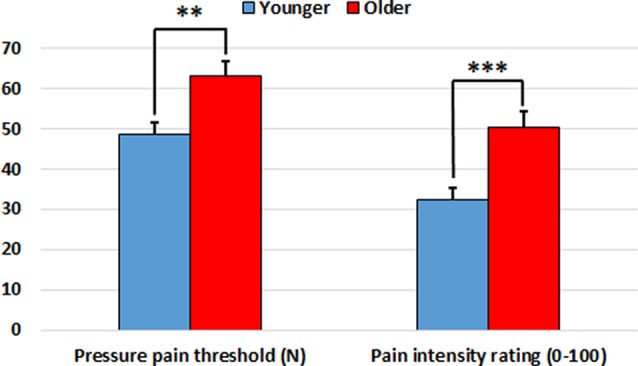
Pressure pain threshold index (in Newtons) and subjective pain rating index (0–100) in the younger and older groups. Older participants showed increased indexes in comparison to younger participants. **p < 0.01, ***p < 0.001.

Older participants showed higher pressure pain thresholds than younger participants on the finger (*t*_(68)_ = 3.51, *p* = 0.001), wrist (*t*_(68)_ = 2.18, *p* = 0.033) and shoulder (*t*_(68)_ = 2.64, *p* = 0.010). Furthermore, older participants gave higher pain intensity ratings than younger participants for the finger (*t*_(68)_ = 3.64, *p* = 0.001), wrist (*t*_(68)_ = 3.33, *p* = 0.001) and shoulder (*t*_(68)_ = 3.72, *p* < 0.001). Accordingly, both the pressure pain threshold index (*t*_(68)_ = 3.22, *p* = 0.002) and the subjective pain rating index (*t*_(68)_ = 3.72, *p* < 0.001) were significantly higher in the older group in comparison to the younger one (see Figure 1 and Table 1).

Also, given the group difference in systolic blood pressure, one-way ANCOVA of the pressure pain threshold index and the subjective pain rating index controlling for systolic blood pressure were conducted. The differences between groups were maintained after controlling for systolic blood pressure (see Supplementary Material).

### Functional Connectivity

**Figure 2:**
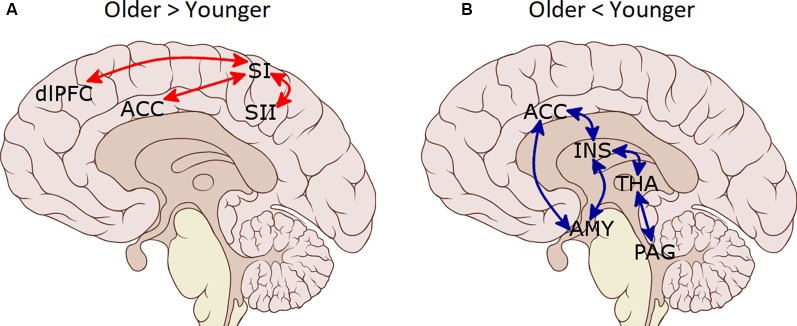
Functional connectivity differences between pain-related regions of interest (ROIs) in the older group as compared to the younger group. **(A)** Increased connectivity in the older group. **(B)** Decreased connectivity in the older group. SI, primary somatosensory cortex; SII, secondary somatosensory cortex; ACC, anterior cingulate cortex; dlPFC, dorsolateral prefrontal cortex; INS, insula; AMY, amygdala; PAG, periaqueductal gray matter; THA, thalamus.

Functional connectivity analyses of the pain-network (Table 3 and Figure 2) showed increased connectivity in the older group compared to the younger one between SI (L) and ACC, SII (R) and dlPFC (R), as well as between ACC and SI (R). Moreover, decreased connectivity in the older group compared to the younger was found between the AMY (R) and bilateral INS, THA (R), ACC and AMY (L); between AMY (L) and INS (R), and bilateral THA; between ACC and bilateral INS; and between PAG and bilateral THA. Regarding the DMN, increased connectivity in the older group compared with the younger group was found between LP (R) and PCC/precuneus (*t*_(68)_ = 2.41, *p* = 0.028).

**Table 3:** Functional connectivity differences derived from pain-network ROI to ROI analyses in older compared to younger groups.

	*T*_(68)_	p-unc	p-FDR
Older > Younger			
SI (L)—ACC	3.38	0.0006	0.0076
SI (L)—SII (R)	3.29	0.0008	0.0100
ACC—SI (R)	2.57	0.0061	0.0394
SI (L)—dlPFC (R)	2.40	0.0095	0.0411
Older < Younger			
INS (R)—AMY (R)	−3.85	0.0001	0.0016
INS (L)—ACC	−3.43	0.0005	0.0065
AMY (R)—AMY (L)	−3.41	0.0005	0.0069
AMY (R)—ACC	−3.01	0.0018	0.0116
AMY (R)—INS (L)	−2.86	0.0028	0.0180
PAG—THA (R)	−2.77	0.0036	0.0239
AMY (R)—THA (R)	−2.76	0.0037	0.0239
INS (R)—AMY (L)	−2.72	0.0041	0.0259
INS (R)—ACC	−2.58	0.0060	0.0259
PAG—THA (L)	−2.54	0.0066	0.0428
AMY (L)—THA (L)	−2.45	0.0083	0.0360
AMY (L)—THA (R)	−2.22	0.0147	0.0478

*SI, primary somatosensory cortex; SII, secondary somatosensory cortex; ACC, anterior cingulate cortex; dlPFC, dorsolateral prefrontal cortex; INS, insula; AMY, amygdala; PAG, periaqueductal gray matter; THA, thalamus; p-unc, p-uncorrected; p-FDR, p-False Discovery Rate*.

### Correlational Analyses

**Figure 3:**
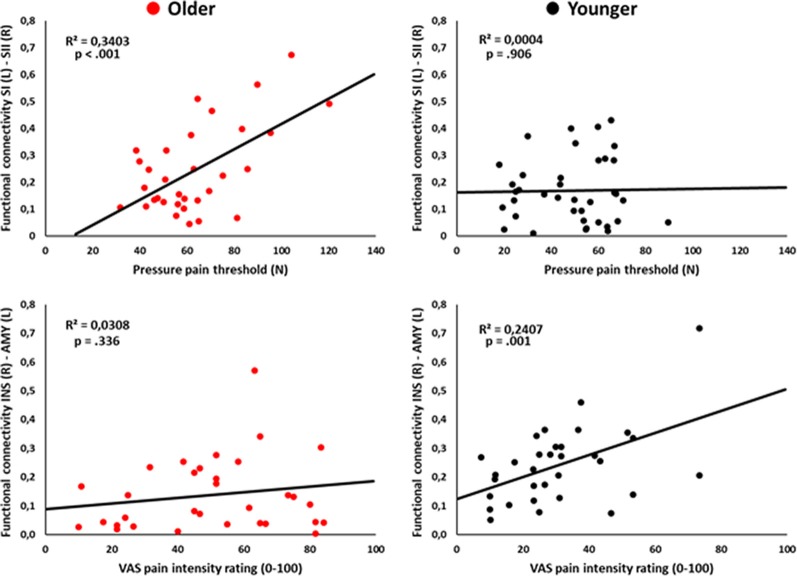
Scatter plots showing the correlation between left primary somatosensory cortex (SI) and right SII functional connectivity with pressure pain thresholds (upper panel), and the correlation between right insula (INS) and left amygdala (AMY) functional connectivity with pain intensity ratings (lower panel) in the older and younger groups.

Pearson’s correlational analyses showed that functional connectivity between SI (L) and SII (R) was positively associated with the pressure pain threshold index in the older group (*r* = 0.58, *p* < 0.001; Figure 3). Also, functional connectivity between the AMY (L) and INS (R) was positively correlated with the subjective pain rating index in the younger group (*r* = 0.51, *p* = 0.001; Figure 3). No-significant correlations regarding PANAS positive scores were found (all *p*s > 0.003).

## Discussion

This study aimed to analyze the age-related changes in spontaneous brain activity to find possible explanations for the increased pain thresholds and lack of pain inhibition that characterizes pain processing in older adults. The analysis of rsFC imaging data and the possible relationship with pressure pain thresholds in older compared to young participants revealed the following main results. First, older participants showed an aberrant hyperconnectivity of the SI with SII and frontal brain areas (dlPFC and ACC) at rest. Second, the hyperconnectivity between somatosensory regions was related to increased pressure pain thresholds in the older group. Third, older participants showed decreased rsFC between brain regions that constitute the brain circuitry defined as the descending pain modulatory system (ACC, INS, AMY, THA, and PAG). Fourth, DMN functioning was rather well preserved in the older participants and differences in connectivity concerning the young group were found only between the LP cortex and PCC/precuneus. Although, connectivity between DMN regions was not related to pain perception changes. The implications of these findings are discussed below.

The main findings from studies on pain sensitivity that have been carried out in humans include an increased threshold and decreased tolerance with advancing age mechanisms (Lautenbacher, 2012; Yezierski, 2012). These results have been interpreted in terms of age exerting opposite influences on the sensory dimension of pain vs. the affective dimension (Yezierski, 2012; Wrobel et al., 2016). We found that older adults showed increased pressure pain thresholds and related pain ratings in comparison to younger adults. Thus, our results support the idea that the pain system in the elderly is activated a little later (Lautenbacher, 2012; Lautenbacher et al., 2017), and therefore when stimulus is detected it is perceived as more intense. Interestingly, pain thresholds were positively associated to SI-SII connectivity in the older participants. This result is in line with previous studies showing that impairment of tactile perception in aging is associated to enlarged cortical representations of the body and cortical excitability of SI (Shaffer and Harrison, 2007; Kalisch et al., 2009; Lenz et al., 2012; Catley et al., 2014). Our results further extend the effects of SI reorganization to the changes produced by aging in pain processing.

Indeed, we found increased connectivity of bilateral SI with ACC, and of left SI with right dlPFC and right SII in older compared to younger participants. SI is a critical component of the nociceptive pathway and is known to encode body location, intensity, and quality of nociceptive stimuli (Bushnell et al., 1999). SII is also related to pain encoding, and like the SI, the magnitude of this activation is significantly related to the perceived intensity of pain (Coghill, 2009). Moreover, it has been shown that SII activity normally depends on SI, rather than thalamic input, providing evidence for serial cortical processing between SI and SII (Pons et al., 1987). Finally, the ACC and dlPFC have been linked to emotional and attentional aspects of pain perception, respectively (Tracey and Mantyh, 2007; Wiech and Tracey, 2009). Overall, our results suggest that in older adults, the rsFC would be characterized by a greater intrinsic information transfer between the SI and associative brain regions involved in further pain processing. Importantly, similar brain reorganizations have been found in other sensory modalities. For instance, compensatory changes in response to complex auditory stimuli from temporal to frontal regions in adults with hearing loss have also been found (Campbell and Sharma, 2013). Similarly, it has been found that the observed decrease in visual memory and visuo-constructive functions seem to be strongly associated with an age-dependent increase of functional connectivity specifically in the temporal lobe (Schlee et al., 2012), which is a key area for visual processing (Conway, 2018). The proposed mechanism to explain these age-effects on sensation and perception is the weakening of cortical inhibition (Dinse et al., 2006; Shaffer and Harrison, 2007; Kalisch et al., 2009; Lenz et al., 2012; Catley et al., 2014; Pleger et al., 2016). The lack of inhibition in sensory areas would lead to enhanced neuronal excitability therein and would favor the enlargement of functional sensory networks (Schlee et al., 2012). Animal studies also support this conclusion since they have consistently shown an age-related decrease of gamma-aminobutyric acid (GABA) inhibitory effectiveness in the auditory (Caspary et al., 1999; Walton et al., 2002), visual (Yang et al., 2008) and somatosensory pathways (Poe et al., 2001). Therefore, decreased cortical inhibition is a likely explanation for the increased rsFC between somatosensory regions found in our study.

Moreover, in agreement with previous studies showing a strong relationship between rsFC of INS and AMY with pain perception (Boly et al., 2007; Proverbio et al., 2009; Ploner et al., 2010), we found that AMY-INS connectivity was related to subjective pain ratings in younger participants. However, no such relationship was found in older adults, which suggests an alteration in the neural networks involved in pain evaluation in this population. Concerning this, we found reduced rsFC within the descending pain modulatory circuitry in older compared to younger participants. This circuitry, including the frontal lobe, ACC, INS, AMY, hypothalamus, and PAG, enables regulation of nociceptive processing and contributes to behavioral (e.g., distraction effect) and opiate analgesia (Valet et al., 2004; Tracey and Mantyh, 2007). Because of its high affinity for opiate binding coupled with high concentrations of endogenous opioids, the PAG is considered a key pain modulating structure (Basbaum and Fields, 1984). The THA is also part of this network (Ab Aziz and Ahmad, 2006), and it has been suggested that functional interactions between PAG and THA are likely to be involved in pain modulation through its connections to the spinal cord dorsal horn (Valet et al., 2004). We found that PAG-THA connectivity was decreased in older participants. Moreover, ACC-INS-AMY connections were also reduced. Therefore, we could conclude that, in our sample, the rsFC between brain regions belonging to the descending inhibitory pain system is reduced in older participants as compared to younger participants. This alteration could have important behavioral implications in situations that require pain modulation. In agreement, older adults show less analgesia through distraction (Zhou et al., 2015a,b), and conditioned pain modulation (i.e., the inhibition of responses to a painful stimulus by another, often spatially distant, pain stimulus) is inversed in older individuals, leading to an enhancement in perceived pain (Lautenbacher, 2012). Furthermore, it has been shown that these changes already begin in middle age when the prevalence of chronic pain is starting to peak (Larivière et al., 2007). Thus, although speculative, this lack of rsFC within the descending pain modulatory circuitry could be related to a lack of pain inhibition and the higher vulnerability of older adults to chronic pain. Furthermore, if during resting-state these areas are less functionally connected, it is also possible that they also show a deficit to reach the activation needed to inhibit pain as good as younger adults do when pain is received. Further studies should explore this possibility.

Finally, we found increased rsFC between LP regions and the PCC/precuneus in older participants in comparison to younger ones. Studies have revealed that increased DMN activity in healthy aging is associated with a higher level of background sensory processing during cognitive tasks (Grady et al., 2006; Li et al., 2007). Our results are in line with these studies, showing increased connectivity within this network in older participants. However, this connectivity was not related to pain perception.

There is a limitation of our study that merit further consideration. Most of the older participants were taking medication. To control for this confounding variable, we replicated the rsFC analyses excluding those subjects who were taking medication that can influence the central nervous system and/or pain perception (see Supplementary Material). Enhanced functional connectivity of SI, SII, and ACC in older participants in comparison to younger ones was replicated. Therefore, it seems that this enhancement of functional connectivity was not influenced by medication intake. Nevertheless, findings regarding the reduced connectivity of the descending pain modulation network in older participants as compared to younger ones were only partially replicated (only INS-AMY connectivity result remained significant). Given that group comparisons remained significant when the statistical correction threshold was lowered from FDR-corrected to *p* < 0.001 uncorrected, it seems probable that the loss of statistical power was due to sample size reduction. Nevertheless, the possible influence of medication in results regarding the descending pain modulation network cannot be completely ruled out. It is well known that antidepressants, as well as anti-inflammatory drugs, may induce anti-nociceptive effects by the interaction with the descending control pain modulatory pathway (Bannister and Dickenson, 2016), but also that antidepressants and anti-inflammatory drugs would provoke a stronger engagement of the descending inhibitory system aiding protection against pain (Bannister and Dickenson, 2016). It seems also relevant to highlight that there is a large amount of literature suggesting a reduced efficacy of the descending pain modulatory system in the elderly (Gibson and Farrell, 2004; Lautenbacher, 2012; Marouf et al., 2014; Lithfous et al., 2019). Hence, further studies must clarify to what extent medication effects may be behind the rsFC changes in older people.

## Conclusion

This study offers new insights into the evolution of cortical networks in normal aging and its relevance to pain perception. The clinical relevance of resting-state networks is notable because the degree of connectivity in these networks predicts individual cognitive, emotional and sensory functions. In our study, older participants showed an abnormal hyperconnectivity of the primary somatosensory area (SI) with other somatosensory and frontal brain regions. This result, together with the positive correlation found between SI-SII functional connectivity and pressure pain thresholds could be interpreted as a compensatory mechanism for the slowed pain processing that seems to accompany aging. Furthermore, older participants showed reduced functional connectivity between key nodes of the descending pain inhibitory pathway. Thus, our results are in line with the suggestion that in aging the pain system is activated lightly later, but, over time, dysfunction of pain modulatory and evaluation processes would lead to increased pain perception (Lautenbacher, 2012). Taken together, these results could explain the greater vulnerability to chronic pain disorders in older individuals. It is important to note that our study was conducted in a very active and healthy sample of older adults. Almost all of them were enrolled in a senior program at our university and none suffered from severe diseases. The alterations found in this study could probably be aggravated in older individuals with worse health conditions, who are less cognitively active. Furthermore, one study showed that brief periods of repetitive sensory stimulation induce plastic changes in SI excitability, and are capable of restoring tactile acuity in older adults to a substantial degree (Pleger et al., 2016). Our results suggest that a similar approach, or other neurorehabilitation techniques (i.e., neurofeedback, transcranial magnetic stimulation, etc.) may be promising avenues to treat pain conditions in an older population.

## Data Availability Statement

The datasets generated for this study are available on request to the corresponding author.

## Ethics Statement

This study was reviewed and approved by Ethics Committee of the Balearic Islands (ref.: IB 3429/17 PI). The participants provided their written informed consent to participate in this study.

## Author Contributions

All the authors have read and approved the article and the procedures used. AG-R, CS, FA, MM, and PM discussed the original design of the experiment. AG-R acquired the data and drafted the original manuscript. AG-R and JT analyzed the data. AG-R, JT, CS, FA, MM, and PM revised the manuscript.

## Conflict of Interest

The authors declare that the research was conducted in the absence of any commercial or financial relationships that could be construed as a potential conflict of interest.

## Footnotes

^1^http://www.fil.ion.ucl.ac.uk/spm/
