# Supplementary material for: Age-Related Changes in Pain Perception Are Associated With Altered Functional Connectivity During Resting State
Source: Front Aging Neurosci. 2020 May 7;12:116. doi: 10.3389/fnagi.2020.00116 (PMC7221150; doi:10.3389/fnagi.2020.00116)
Supplement: Supplementary file 1 [file Data_Sheet_1.pdf]

Supplementary material

Pressure pain thresholds ANCOVA

One-way ANCOVA of the pressure pain threshold index controlling by systolic blood pressure yielded a main group effect ($F(1,69)=5.471$, $p=.022$, $\eta p2=.075$) and a main systolic blood pressure effect ($F(1,69)= 4.097$, $p=.047$, $\eta p2=.058$). The corrected mean values reflected a higher pain threshold in older (63.21 ± 19.42) than in younger (48.46 ± 19.30) groups. Furthermore, one-way ANCOVA of the subjective pain rating index controlling by systolic blood pressure showed a main group effect ($F(1,69)= 9.543$, $p=.003$, $\eta p2=.125$). The corrected mean values indicated a higher rating index in older (49.04 ± 20.84) than in younger (33.11 ± 20.71) groups.

Functional connectivity analyses controlling by medication intake

In order to control for the possible influence of medication intake in our results, we have replicated the functional connectivity analyses excluding those subjects who were taking any medication that could affect central nervous system and/or pain processing (antidepressant, anxiolytic, anti-inflammatory and anti-hypertensive). Hence, 37 young adults (out of 38) and 18 older adults (out of 32) enter in this sub-analysis. Functional connectivity differences derived from pain-network ROI to ROI analyses in older compared to younger groups were:

	T(53)	p-unc	p-FDR
Older > Younger			
SI (L) – ACC	3.38	.0006	.0077
SI (L) – SII (R)	3.85	.0001	.0016
ACC – SI (R)	2.85	.0028	.0183
SI (L) – SI (R)	2.36	.0105	.0453
SI (L) – SII (L)	2.21	.0153	.0496
Older < Younger			
INS (R) – AMY (R)	-3.54	.0004	.0046
INS (L) – ACC	-2.08	.0204	.1324
AMY (R) – AMY (L)	-2.06	.0215	.0969
AMY (R) – ACC	-2.64	.0051	.0334
AMY (R) – INS (L)	-2.17	.0167	.1324
PAG – THA (R)	-2.07	.0211	.2237
AMY (R) – THA (R)	-1.78	.0395	.1713
INS (R) – AMY (L)	-2.16	.0170	.1103
PAG – THA (L)	-1.85	.0344	.2804
AMY (L) – THA (L)	-1.74	.0431	.2804
AMY (L) – THA (R)	-2.04	.0224	.1453

In comparison with the main functional connectivity results, the results for Older adults > Younger adults were very similar. Only the SI(L) – dlPFC(L) connectivity disappear. Moreover, two new connectivities (SI(L) – SI(R) and SI(L) – SII(L)) were obtained. Hence, the abnormal connectivity of the primary somatosensory area with other somatosensory and frontal brain regions in older participants was replicated.

Regarding results for Older adults < Younger adults, only INS(R) – AMY(R) and ACC – AMY (R) connectivities remained as statistically significant. However, all other significant differences found in the main results (except for INS (R) – ACC), were replicated if the statistical

correction threshold was lowered from FDR-corrected to $p<.001$ uncorrected. Hence, in this sub-analysis, part of the results regarding the reduced functional connectivity between key nodes of the descending pain modulatory circuitry in older participants is not showed with the same statistical power.
